# Rapid assessment of faecal egg count and faecal egg count reduction through composite sampling in cattle

**DOI:** 10.1186/s13071-019-3601-x

**Published:** 2019-07-16

**Authors:** Laura Rinaldi, Alessandra Amadesi, Elaudy Dufourd, Antonio Bosco, Marion Gadanho, Anne Lehebel, Maria Paola Maurelli, Alain Chauvin, Johannes Charlier, Giuseppe Cringoli, Nadine Ravinet, Christophe Chartier

**Affiliations:** 1 Department of Veterinary Medicine and Animal Production, University of Napoli Federico II, CREMOPAR, Napoli, Italy; 2 BIOEPAR, INRA, Oniris, 44307 Nantes, France; 3 Kreavet, Hendrik Mertensstraat 17, 9150 Kruibeke, Belgium

**Keywords:** Gastrointestinal strongyles, Mini-FLOTAC, FECRT, Pooled faecal samples, Cattle

## Abstract

**Background:**

Faecal egg counts (FEC) and the FEC reduction test (FECRT) for assessing gastrointestinal nematode (GIN) infection and efficacy of anthelmintics are rarely carried out on ruminant farms because of the cost of individual analyses. The use of pooled faecal samples is a promising method to reduce time and costs, but few studies are available for cattle, especially on the evaluation of different pool sizes and FECRT application.

**Methods:**

A study was conducted to assess FEC strategies based on pooled faecal samples using different pool sizes and to evaluate the pen-side use of a portable FEC-kit for the assessment of FEC on cattle farms. A total of 19 farms representing 29 groups of cattle were investigated in Italy and France. On each farm, individual faecal samples from heifers were collected before (D0) and two weeks after (D14) anthelmintic treatment with ivermectin or benzimidazoles. FEC were determined individually and as pooled samples using the Mini-FLOTAC technique. Four different pool sizes were used: 5 individual samples, 10 individual samples, global and global on-farm. Correlations and agreements between individual and pooled results were estimated with Spearman’s correlation coefficient and Lin’s concordance correlation coefficients, respectively.

**Results:**

High correlation and agreement coefficients were found between the mean of individual FEC and the mean of FEC of the different pool sizes when considering all FEC obtained at D0 and D14. However, these parameters were lower for FECR calculation due to a poorer estimate of FEC at D14 from the faecal pools. When using FEC from pooled samples only at D0, higher correlation and agreement coefficients were found between FECR data, the better results being obtained with pools of 5 samples. Interestingly, FEC obtained on pooled samples by the portable FEC-kit on-farm showed high correlation and agreement with FEC obtained on individual samples in the laboratory. This field approach has to be validated on a larger scale to assess its feasibility and reliability.

**Conclusions:**

The present study highlights that the pooling strategy and the use of portable FEC-kits on-farm are rapid and cost-effective procedures for the assessment of GIN egg excretion and can be used cautiously for FECR calculation following the administration of anthelmintics in cattle.

## Background

Gastrointestinal nematode (GIN) parasites, also known as gastrointestinal strongyles (Strongylida, Trichostrongyloidea), are amongst the most important production-limiting pathogens of grazing ruminants in Europe and globally (http://www.discontools.eu) [1]. The negative impact of GIN on livestock farms is further exacerbated by the escalating spread of anthelmintic resistance (AR) [2], a phenomenon under the attention of the scientific community and stakeholders as demonstrated by several European initiatives including the COST Action COMBAR (COMBatting Anthelmintic Resistance in Ruminants; https://www.combar-ca.eu/; CA16230) recently launched to coordinate research on the control of AR in helminth parasites of ruminants.

One of the options to make GIN control practices more sustainable is to lower drug application frequency by targeting treatment (TT) to the whole group of animals when infection is high while preserving a pool of unexposed parasites in refugia as free-living stages [3]. Diagnosis of gastrointestinal helminth infection is mainly based on the detection of worm eggs through faecal egg counts (FEC) [1]. The TT approach requires a relevant method (e.g. FEC) that indicates the worm burden of a given group despite the over-dispersed distribution of parasites within a group of animals [4]. Furthermore, there is an urgent need to obtain better information on the AR status in Europe and FEC are required to estimate anthelmintic efficacy/resistance by the faecal egg count reduction test (FECRT) [5].

To perform this test, the ideal group size is around 10 to 15 animals [6]. However, the cost of individual FEC is too high for ruminant farmers and makes veterinarians reluctant to increase FEC-based investigations [7]. As a result, on most ruminant farms, faecal diagnosis is rarely carried out, if at all [2]. A more regular employment of copromicroscopic monitoring of worm egg excretion could be facilitated by reducing the number of individual FEC analyses through the use of composite (pooled) faecal samples in which equal amounts of faeces from several animals are mixed together and a single FEC is determined from the mixture as a proxy of the group mean FEC.

Several studies have been performed in sheep comparing mean individual counts to pooled counts using different pool sizes, ranging from three to ten samples, and different analytic sensitivities of the FEC technique, ranging from 10 to 50 eggs per gram (EPG) of faeces [8–12]. These studies indicated that pooling ovine faecal samples was a reliable procedure for assessing GIN FEC taking into account the level of FEC, the pool size and the analytical sensitivity of the method [11].

Less is known about faecal pooling in cattle. Ward et al. [13] in Australia showed a good agreement between mean individual counts (*n* = 10) and mean composite counts (two pools of five), and George et al. [14] in the USA successfully tested the single pooling from a group of animals ranging from 9 to 19 individuals (mean number of 15.7). However, these two studies were based either on a composite sample made from two pools of five individual faecal samples or on a single pool of all the individual samples and did not investigate the effect of different pool sizes on the FEC estimation.

Besides pooling, field-applicable kits allowing on-farm implementation of FEC with easy-to-use devices to quickly analyze pooled samples are needed by the new generation of veterinarians and farmers to quantify helminth infection, anthelmintic efficacy and AR. Recently, portable FEC kits combined with a mobile phone application have been developed for image capture and specific worm egg quantification in horses and humans [15–17].

In order to further improve and evaluate the rapid and cost-effective evaluation of FEC and related FECR in cattle, field studies were conducted in order to: (i) further evaluate strategies to assess FEC based on pooled faecal samples (using different pool sizes); and (ii) develop and evaluate a portable FEC-kit in order to perform pooled FEC on-farm.

## Methods

### Study design and sampling

Between June and October 2017, field trials were conducted on a total of 19 cattle farms located in Italy and France. Specifically, in Italy 10 beef cattle farms were included and selected in the Campania and Basilicata regions (southern Italy); cattle were crossbreeds (Limousine, Podolica, Marchigiana). In France, 9 farms were included and selected in Normandy and Brittany regions (north-western France); they were Holstein or Normande breed dairy farms. In both countries, the farms were initially randomly chosen within the selected regions and then the selection was mainly driven by the availability of the farmer and the presence of GIN positive cattle.

Overall on each farm, individual faecal samples (20 g at least) from first or second grazing season heifers (aged from 6 to 20 months) were collected before (D0) and two weeks after (D14) anthelmintic treatment, i.e. ivermectin (IVM, injectable solution, 0.2 mg/kg of body weight) or albendazole/fenbendazole (ABZ/FBZ, oral suspension, 7.5 mg/kg of body weight). When the number of heifers on a given farm was much higher than 20 and thus exceeded the average value met on most farms, animals were split into similar groups of 10/20 animals and assigned a different treatment.

**Fig. 1:**
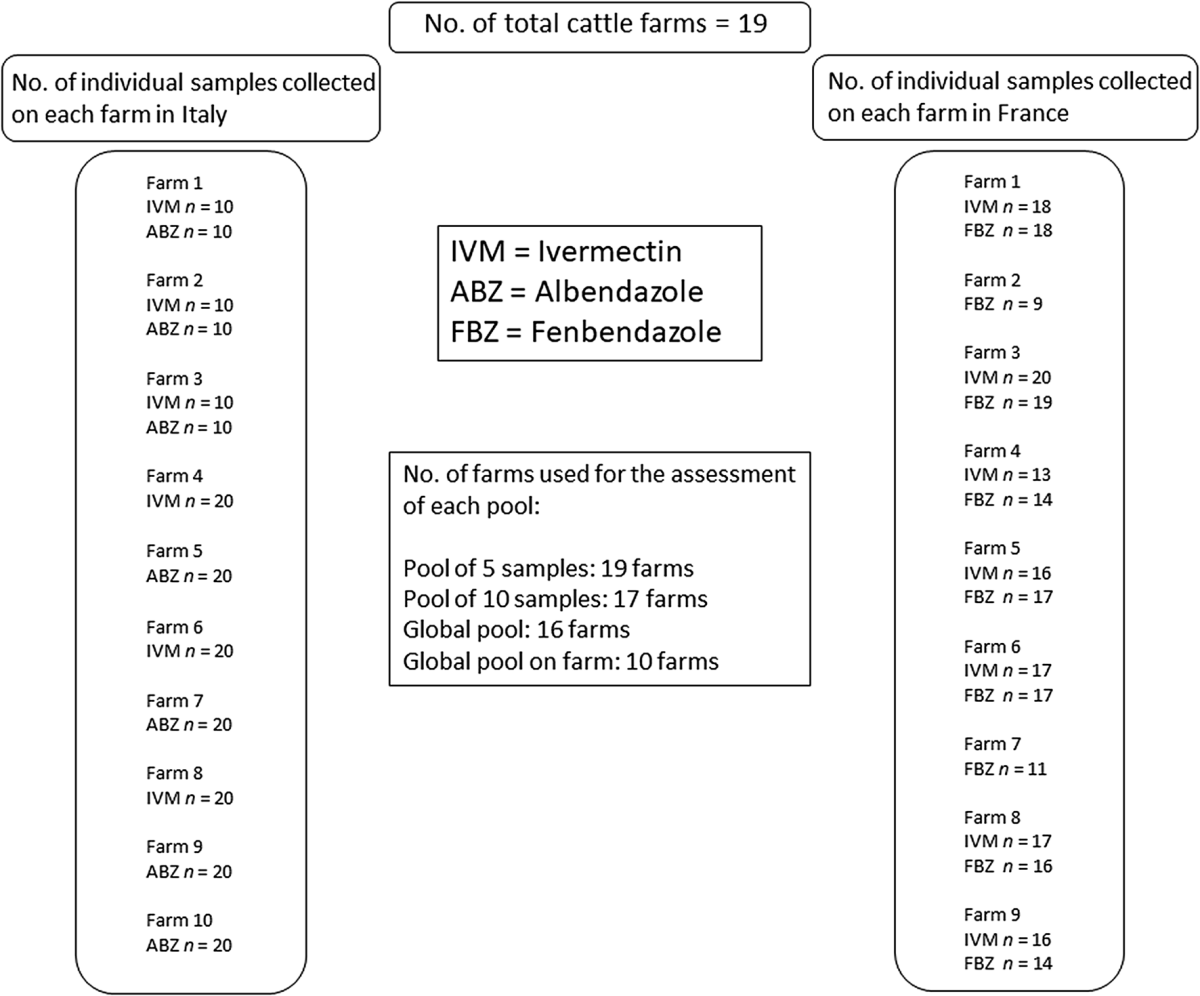
The number of Italian and French cattle farms, individual faecal samples and pools used for the study. *Abbreviations*: ABZ, albendazole; FBZ, fenbendazole; IVM, ivermectin

In Italy, the animals were divided into 2 groups of 10 animals (one group treated with IVM and one with ABZ) on 3 farms; on 3 other farms, 20 cattle were treated with IVM and on 4 farms 20 cattle were treated with ABZ. Similarly, in France, on 2 farms the animals were divided into 2 groups of 18–20 animals, with on each farm one group treated with IVM and the other with FBZ; on 6 other farms, animals were divided into 5 and 6 groups of 11 to 18 animals, respectively; within each farm the groups were assigned to a treatment with either IVM or FBZ. On one farm, a single group of 9 animals was treated with FBZ. Therefore, a total of 29 groups of cattle were available for evaluating the relationship between mean FEC of the individuals and the composite samples, 13 groups (6 treated with IVM and 7 with ABZ) in Italy and 16 groups (7 treated with IVM and 9 with FBZ) in France. The total number of cattle farms, individual faecal samples and pools used for the study are provided in Fig. 1.

### Preparation of pooled samples and parasitological analysis

At D0 and D14, bovine faecal samples were analyzed both individually and as pooled samples using the Mini-FLOTAC technique with a detection limit of 5 eggs per gram (EPG) of faeces, using a sodium chloride flotation solution (FS2, specific gravity = 1.200) [18]. Three different pool sizes were used when possible (5 or 10 individual samples, global pooling) according to the protocol described in Rinaldi et al. [12] and Kenyon et al. [11]. Briefly, each sample was labelled, thoroughly homogenized, individually examined and then composite (pooled) samples were prepared taking approximately 5 g of each sample with the collector of the Fill-FLOTAC [18].

It should be noted that the predefined pool sizes of 5 and 10 could not always be met at both D0 and D14 due to some practical constraints such as the exact number of animals in the group and an insufficient amount of faeces to perform the analysis of each pool. The actual pool sizes (number of animals from which an individual faecal sample was included) ranged from 3 to 6 for pools of 5 and from 6 to 10 for pools of 10. The global pool was made from all the individuals whatever the group size (ranging from 9 to 20). At D0 and D14, the same animals were sampled and the same pools were prepared. When one sample was missing in a given pool, the corresponding sample was withdrawn before individual FECs were averaged.

### FECR on-farm

A portable FEC-kit was developed in order to perform pooled FEC on-farm. The kit consisted of 2 Fill-FLOTAC (for sample collection and weighing, homogenization, filtration and filling) and 2 Mini-FLOTAC devices [18], the flotation solution (FS2) and a portable (hand-held) microscope with batteries (Celestron, Torrance, CA, USA) for use on-farm. This portable FEC-kit was used on 10 farms to assess a global pool FEC at D0 and/or D14. Briefly, a single pooled sample was prepared taking 5 g of faeces from all individual samples using Fill-FLOTAC and then thoroughly mixed with a spatula in a large beaker. From this pool (90–100 g), a single sample of 5 g was taken by the Fill-FLOTAC and analyzed using the Mini-FLOTAC technique [18] combined with the reading by a senior researcher under the hand-held microscope.

### Coprocultures

For each of the 29 groups of cattle, a pooled faecal culture was performed at D0 and D14, following the protocol described in MAFF [19]. Developed third-stage larvae (L3) were identified using the morphological keys proposed by van Wyk & Mayhew [20]. Identification and percentages of each nematode genera were conducted on 100 L3; if a sample had 100 or less L3 present, all larvae were identified. So, on the total number of larvae identified, it was possible to give the percentage of each genus.

### Statistical analysis

The mean FEC of individual and pooled samples were calculated as the arithmetic mean. Correlations between the different measures of FEC were assessed by Spearman’s rho correlation coefficient (*r*_s_), the associated 95% confidence interval (CI) and *P*-value. Moreover, Lin’s concordance correlation coefficients (CCC) and the corresponding 95% CI were calculated to quantify the agreement between the analysis from individual samples and each pool size (including those performed on-farm). Like a correlation, CCC ranges from − 1 to 1, with perfect agreement at 1. The strength of agreement was classified as poor, moderate, substantial or almost perfect for CCC values < 0.9, 0.90–0.95, 0.95–0.99 or > 0.99, respectively [21].

When examining individual samples, the FECR (%) was calculated according to the formula: FECR (%) = [1 − (arithmetic mean of post treatment individual FECs/ arithmetic mean of pre-treatment individual FECs)] × 100. For each size of pooled samples (5, 10, global), the FECR (%) was calculated as the percent reduction in pooled FEC at D14 compared to corresponding pooled FEC at D0: FECR (%) = [1 − (arithmetic mean of post treatment pooled FECs/ arithmetic mean of pre-treatment pooled FECs)] × 100, the number of pools ranging from 1 to 4. Spearman’s *r*_s_ and Lin’s CCC were calculated as above between FECR (%) from individual and pooled samples. In addition, a further correlation analysis (*r*_s_ and CCC) was done for the calculation of FECR (%) using a “mixed approach”, i.e. using FEC on D0 based on pooled samples and FEC on D14 based on individual samples.

The following criterion was used for defining reduced efficacy: FECR < 95% and lower limit of 95% confidence interval < 90% [6].

The level of significance was set at a *P*-value < 0.05 for all tests. All statistical analyses were performed using GraphPad Prism v.5 (Graph Pad Software, San Diego, CA, USA) and SPSS Statistics v.23 (IBM, Armonk, NY, USA).

## Results

### FEC in individual and composite samples

A total of 200 individual samples were analyzed in Italy and 252 in France. When calculated from individual samples, the mean GIN FEC at D0 and FECR (%) varied between 9.2–359 EPG and 73.3–100%, respectively, providing reasonable variation in FEC and FECR (%) values to be tested in the pooling strategy.

**Fig. 2:**
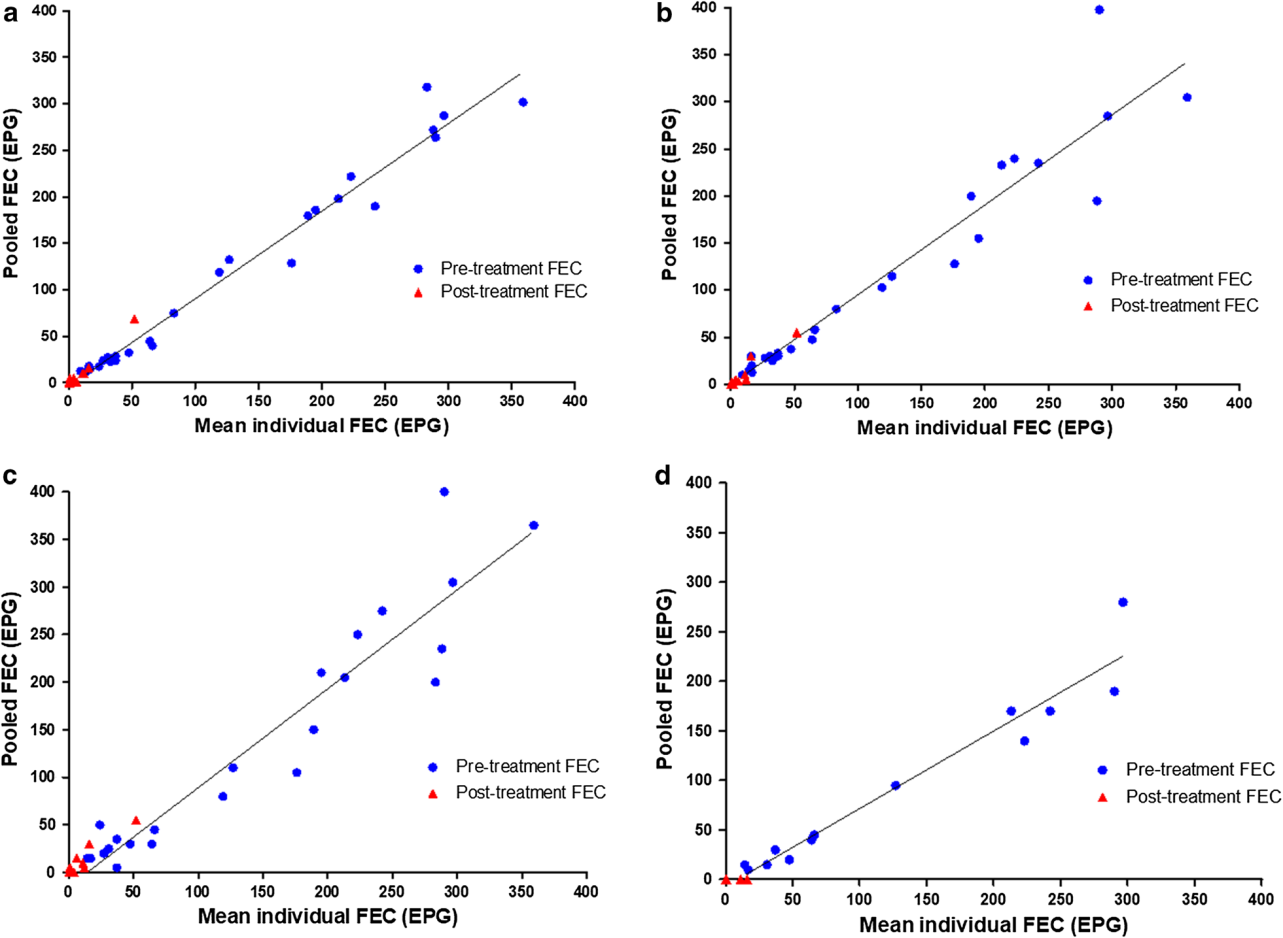
The correlation in FEC (pre-treatment and post-treatment) based on the examination of individuals and pools of 5 (**a**), 10 (**b**), global pool (**c**) and global pool analysed directly on-farm (**d**) in Italy and France

The correlation and the agreement between FEC results from individual means and pool means are reported in Table 1 and Fig. 2. Overall, the FEC results of pooled samples strongly correlated with those of individual samples regardless of the pool sizes. When focusing on FEC values at D0 or D14, i.e. FEC ranging between 5–400 EPG and 0–69 EPG, respectively, Spearman’s *r*_s_ values were notably lower for D14 FEC values.

**Table 1:** Spearman’s rho correlation coefficient (*r*_*s*_) and Lin’s concordance correlation coefficients (CCC) between FEC from individual and pooled samples according the pool size and the FEC values (whole, D0 or D14) and between FECR(%) from individual samples and FECR(%) from individual samples at D14 and pooled samples at D0 according the pool size

Pool size	No. of pools	*r*_s_	95% CI	CCC	95% CI
Faecal egg count					
Pool of 5 samples	58	0.98	0.95–0.99	0.99	0.98–0.99
Pool of 10 samples	42	0.97	0.92–0.99	0.97	0.94–0.98
Global pool	58	0.95	0.91–0.97	0.97	0.95–0.98
Global pool on-farm	26	0.94	0.82–0.98	0.93	0.88–0.96
Pool of 5 samples (D0)	29	0.98	0.93–0.99	0.98	0.96–0.99
Pool of 10 samples (D0)	21	0.98	0.90–1.00	0.94	0.86–0.98
Global pool (D0)	29	0.91	0.75–0.97	0.95	0.90–0.98
Pool of 5 samples (D14)	29	0.84	0.64–0.94	0.96	0.93–0.97
Pool of 10 samples (D14)	21	0.79	0.51–0.92	0.95	0.90–0.98
Global pool (D14)	29	0.69	0.39–0.86	0.94	0.89–0.97
Faecal egg count reduction					
Pool of 5 samples	29	1.00	0.99–1.00	0.97	0.95–0.98
Pool of 10 samples	21	0.88	0.72–0.95	0.82	0.65–0.91
Global pool	29	0.80	0.62–0.91	0.82	0.70–0.90
Global pool on-farm	13	0.68	0.20–0.90	0.89	0.85–0.91

The overall level of agreement between the FEC from individual and pool means was substantial for pool of 5 (CCC = 0.99, *P* < 0.001), pool of 10 (CCC = 0.97, *P* < 0.001) or global pool (CCC = 0.97, *P* < 0.001). When considering results separately for D0 or D14, the agreement was substantial for pool of 5 (CCC = 0.98, *P* < 0.001 and CCC = 0.96, *P* < 0.001, respectively) and moderate for pool of 10 (CCC = 0.94, *P* < 0.001 and CCC = 0.95, *P* < 0.001, respectively) or global pool (CCC = 0.95, *P* < 0.001 and CCC = 0.94, *P* < 0.001, respectively).

Regarding the diagnosis directly on-farm including D0 and D14 values, results showed a high correlation (*r*_s_ = 0.94, *P* < 0.001) and a moderate level of agreement (CCC = 0.93, *P* < 0.001).

The correlation between FECRs resulting from individual and composite samples showed *r*_s_ values significant but moderate for pools of 5 samples (*r*_s_ = 0.80, *P* < 0.001), 10 samples (*r*_s_ = 0.77, *P* < 0.001) and global pools (*r*_s_ = 0.67, *P* < 0.001). Similarly, CCC values indicated a poor and decreasing level of agreement for pool of 5 samples (CCC = 0.74; *P* < 0.001) and global pool (CCC = 0.49, *P* < 0.001). When considering a mixed determination of FECR using FEC at D0 based on pooled samples and FEC at D14 based on individual samples (Table 1), higher values were obtained both for Spearman’s correlation coefficients and for CCC values. Specifically, the better results were obtained with pools of 5 samples (*r*_s_ = 1.00, *P* < 0.001; CCC = 0.97, *P* < 0.001) and the worst with the global pool (*r*_s_ = 0.80, *P* < 0.001; CCC = 0.82, *P* < 0.001). Data were less available for global pool on-farm and indicated low correlation value (*r*_s_ = 0.68, *P* < 0.001) and a poor level of agreement (CCC = 0.89, *P* < 0.001).

### Coprocultures

In Italy, the following GIN genera were detected at D0 (pre-treatment): *Cooperia* (41%), *Trichostrongylus* (20%), *Oesophagostomum* (18%), *Ostertagia* (11%) and *Haemonchus* (10%); at D14 (post-treatment) all samples were negative for GIN larvae. In France, the following GIN genera were detected at D0 (pre-treatment): *Cooperia* (88%) and *Ostertagia* (12%). At D14 (post-treatment), the following GIN genera were detected: *Cooperia* (99%) and *Ostertagia* (1%) on the farms treated with IVM, whilst very few numbers of *Cooperia* and *Ostertagia* were found at D14 on farms treated with FBZ.

## Discussion

Diagnosis of GIN infections by the examination of individual faecal samples, although simple and effective, remains expensive and time-consuming which hampers widespread adoption by farmers. Over the last decade, thanks to the development of new diagnostic approaches and the improvement of the existing ones, considerable progress has been made to improve the performance (e.g. increasing the analytic sensitivity, accuracy and precision) of FEC and FECR in livestock.

However, to increase user-friendliness and uptake of the FEC and FECR by veterinarians and farmers, portable kits are required to make rapid decisions on the need to treat or to determine whether anthelmintics are effective [1].

In addition, promising results have been obtained in pilot studies using pooled faecal samples to decrease the workload and cost of conducting FEC in sheep and cattle [11, 12, 14]. Moreover, in all these studies, as well as in a recent study on a comparison between different FEC methods (McMaster, Wisconsin and Mini-FLOTAC) in four different livestock hosts (cattle, sheep, llamas and horses) [22], the good performance of Mini-FLOTAC was emphasized especially when high accuracy is important, such as when measuring FECR.

In the light of these findings, in the present study a practical approach was developed for a rapid and accurate assessment of GIN infection intensity before and after anthelmintic treatment in cattle in Italy and France. The experiment was conducted in parallel in two countries where the susceptibility of GIN could vary as it has been previously mentioned for small ruminants [12] but also encompassing potential variation in the laboratory settings where the tests were performed.

The present study provides new insights into standardization of FEC and FECRT on pooled faecal samples by comparing different pool sizes (five samples, ten samples and global) in cattle and the evaluation of a portable kit to perform pen-side FEC.

High correlation and agreement coefficients (Spearman and Lin) were found between the mean of individual FECs and the mean of FECs of three different pool sizes (five samples, ten samples and global) when considering all FEC obtained at D0 and D14. Values were in the same range for the different pools (0.95 to 0.98 for *r*_s_ and 0.97 to 0.99 for CCC) and indicated that any pooling strategy was efficient. However, when focusing on the lowest FECs, i.e. those obtained 14 days after anthelmintic treatment, correlations were noticeably lower suggesting a poorer estimation of FEC through pooling, due to a lot of zero data. These poor estimates of FEC at D14 were responsible for a poor FECR calculation.

In contrast, when FEC determination at D14 was based on individual faecal samples, noticeably higher correlation/agreement values were found for FECR, particularly for a pool of five samples. Our results globally confirm the previous data on pooled FEC/FECR obtained in sheep by Kenyon et al. [11] and Rinaldi et al. [12] and in cattle by Ward et al. [13] and George et al. [14] with different pooling strategies (pools of 5, 10 or 20; global pool of 9–19 animals). In the study of George et al. [14] involving 14 groups of cattle, the mean individual FEC ranged from 82 to 671 and from 0 to 210 EPG for pre-treatment and post-treatment sampling, respectively whereas the FECR (%) ranged from 53.1 to 100. The authors found very high correlation (*r*_s_ = 0.92) and agreement (CCC = 0.95) of FECR (%) between individual and global pooling sampling (9–19 animals per pool). Such distributions in mean individual FEC and in FECR (%) have not been found in the context of the French and Italian cattle production. Kenyon et al. [11] pointed out the importance of the EPG level and the EPG aggregation at D0 for the use of pooled faeces for FECR.

Interestingly, FECs obtained on pooled samples by the portable FEC-kit on-farm showed high correlation and agreement with FECs obtained on individual samples in the laboratory. This field approach has to be validated on a larger scale to assess the feasibility and reliability of FECR calculation on-farm.

The present study also confirmed the findings by Geurden et al. [23] with the full efficacy of ivermectin on cattle farms in Italy and the lack of efficacy on some farms in France.

## Conclusions

The present study highlighted that the pooling strategy and the use of a portable FEC-kit on-farm are rapid and cost-effective procedures for the assessment of GIN egg excretion and can be used cautiously for FECR calculation following administration of anthelmintics in cattle. The use of improved FEC and FECR together with harmonization of study design and interpretation [14, 24] would allow field surveys to be conducted on a larger scale than today. It would also promote uptake of diagnostic procedures by veterinary practitioners in order to fill knowledge gaps in the burden of GIN infection and the efficacy of anthelmintics at both the European and global scale. For these reasons, the development of an automated system for reading and counting eggs based on the Mini-FLOTAC technique in the veterinary field is in progress. It uses remote support tools to assist veterinarians and farmers to optimize control strategies so that evidence-based parasite control strategies for livestock can be effectively implemented in the future.

## Data Availability

All data generated or analysed during this study are included in this published article. The datasets used and/or analysed during the present study available from the corresponding author upon reasonable request.

## Abbreviations

GIN: gastrointestinal nematode
GI strongyles: gastrointestinal strongyles
D0: pre-treatment
D14: post-treatment
IVM: ivermectin
ABZ: albendazole
FBZ: fenbendazole
FEC: faecal egg count
FECR: faecal egg count reduction
FECRT: faecal egg count reduction test
AR: anthelmintic resistance
TT: targeted treatment
*r*_s_: Spearman’s rho correlation coefficient
CI: confidence interval
CCC: Lin’s concordance correlation coefficients
